# Ovarian cancer in Morocco: Time to act is now

**DOI:** 10.1016/j.gore.2021.100857

**Published:** 2021-09-03

**Authors:** Khalid El Bairi, Ouissam Al Jarroudi, Said Afqir

**Affiliations:** Department of Medical Oncology, Mohammed VI University k.elbairi@ump.ac.ma, Oujda, Morocco; Faculty of Medicine and Pharmacy, Mohammed I^st^ University, Oujda, Morocco

**Keywords:** Ovarian cancer, Morocco, Global oncology, OVANORDEST

## Abstract

• Ovarian cancer seems is a neglected cancer in Morocco.
• No publications that impact clinical practice were published in the last decade.
• In this editorial, we provide our vision to develop this ignored area of gynecologic oncology.

Bibliometrics, informetrics, scientometrics and others are influential analytical tools of scholarly literature. They hold promise for building accurate governmental policies to develop and support evidence-based decision-making and disease prevention as well as control strategies to better improve patients’ care through the use of the findings of scientific research (Ismail et al., 2012, Thompson and Walker, 2015). In Morocco, updated results of the GLOBOCAN initiative (available at: https://gco.iarc.fr/) shows ovarian cancer (OC) as the third incident gynecological cancer and also the third in terms of mortality from malignancies of the reproductive tract. We have recently conducted and published a bibliometric analysis of research on OC that covered 118 years for the first time in Morocco (El Bairi et al. 2021c). Our report raised several concerns about clinical research on such an aggressive woman’s cancer. First, no specific clinical articles on OC were found indexed on Pubmed. Only two translational publications on breast cancer included some patients with OC. Remarkably, the majority of the literature that we analyzed was dominated by case reports, namely low-impact literature on oncology practice and to inform health policy formulation. Therefore, a significant “patient impact factor” (i.e., research with impact on patient care) has not been achieved yet in our setting. The H-index of Moroccan researchers working in this field was very low (<10). This suggests that no expertise in clinical research on OC has been developed in the last decade. Moreover, international collaboration was only noticed for research involving preclinical studies and review articles. The number of publications per million of inhabitants was lower than 1 when exploring research productivity on OC standardized by the population size. Likewise, this trend was also markedly observed for the number of articles per new cases of OC and per GDP. The engagement of women in OC research was under-represented. They were largely dominating first author positions but rarely listed as leading and supervising principal investigators. In summary, no research on epidemiological, clinical, and pathological aspects of OC has been published. This poor production and visibility of OC research in Morocco may be attributed to several well-known factors in countries with limited resources. This encompasses the limited funding support and research initiatives in gynecologic oncology, and the inadequate training of the health care professionals to conduct clinical research. Furthermore, Morocco has no medical journals that are indexed on Pubmed/Medline as compared to some North-African countries such as Egypt and Tunisia. Thus, publishing national scientific production in medicine is challenging. Historically, the “Maroc Médical/al-Maghrib al-tibbi” was the only local journal that was indexed on Pubmed between 1945 and 1986, but was discontinued later. Therefore, the development of a national journal in the field of oncology is urgently needed. Another major barrier is the absence of “Gynecologic Oncology” as an independent specialty. OC patients are managed by gynecologists and obstetricians with no formal training in cancer care; therefore, clinical research on OC is significantly affected by the lack of expertise.

We developed an individual initiative, called OVANORDEST (“OVAire dans le NORD-EST” (ovarian cancer in North-East of Morocco)) studies that aims to develop clinical research on OC for the first time in Morocco. Our main goal is to implement research in OC based in our country, expected to enhance clinical research in this neglected area. Our vision (Fig. 1) encompassed three key milestones over 14 years as follows:

**Fig. 1:**
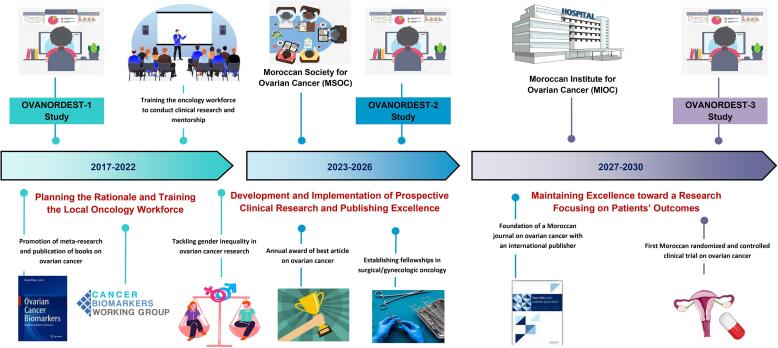
Global overview of OVANORDEST project.

**Step 1 (2017–2022): Planning the Rationale and Training the Local Oncology Workforce**

– Conducting a bibliometric analysis of the national scientific production on OC (as commented).
– Promoting the publication of books, reviews, and systematic reviews and *meta*-analyses on emerging topics in gynecologic oncology with a particular focus on OC.
– Preparing special issues for international *Medline*-indexed journals to foster international collaboration and increase the visibility of national oncologists.
– Initiating OVANORDEST-1 study: a retrospective study that is expected to provide the rationale for future prospective studies. The cohort will collect data from archived patients’ files to present an overview of epidemiology, clinic-pathologic patterns, and survival outcomes of women with OC. It will also investigate various inexpensive and potential prognostic and predictive markers for OC. This would be the first study of this type, and is intended to support cancer registration, and real-life data.
– Supporting oncology residents to develop expertise in the management of OC through multidisciplinary team meetings, fellowships and participation in masterclasses of international societies such as *ESMO*, *ESGO*, *IGCS*, and *ASCO*, and others.
– Training the workforce of local oncologists to conduct research in medical oncology.
– Building partnerships with the “Institut de Recherche sur le Cancer”.
– Improving the role of women oncologists in clinical research on OC to limit gender inequality and increasing the visibility and engagement of junior female researchers on OC.
– Developing a working group on cancer biomarkers to develop national and international collaborations on OC.
– Joining international initiatives and coalitions on OC such as *Global Ovarian Cancer Charter.*

**Step 2 (2023–2026): Development and Implementation of Prospective Clinical Research and Publishing Excellence**

– Initiating OVANORDEST-2 study: a prospective observational trial to validate the findings of our earlier feasibility study and provide high-quality data for biomarker research with prior hypothesis testing.
– Organizing an annual best research article award and conferences on OC, summer schools, and masterclasses on research methods in oncology.
– Supporting national innovative cancer research projects on OC.
– Foundation of the *Moroccan Society for Ovarian Cancer* (MSOC) and development of a Moroccan oncology journal with an international publisher.
– Enhancing policy formulation and aid decision-makers to appropriately invest in OC in Morocco and address evidence-based unmet needs.
– Mentoring undergraduate medical students to encourage them to pursue a career in medical oncology and clinical research and establishing paid research internships for PhD and MD students at our department of medical oncology.
– Raising awareness and empowering patients’ advocacy for OC in Morocco.
– Exploring funding opportunities of international organizations such as the *Conquer Cancer Foundation.*
– Building partnerships with international societies to set up fellowship programs in surgical oncology and gynecologic oncology for Moroccan young general surgeons and gynecologists to develop expertise in OC management.

**Step 3 (2027–2030): Maintaining Excellence Toward a Research Focusing on Patients’ Outcomes**

– Initiating OVANORDEST-3 study: development and prospective validation of an inexpensive score for prognostic classification of OC patients.
– Foundation of the *Moroccan Institute for Ovarian Cancer* (MIOC): a national hospital and institution that is expected to deliver precision medicine and best of care to Moroccan women with OC, as a national and regional reference hub. The institute will also conduct clinical trials and translational research.
– Launching a Moroccan multicenter randomized and controlled clinical trial on OC.
– Maintaining clinical research and delivering improved supportive care for OC patients.

To date, we could prepare two journal special issues, a book, review articles (El Bairi and Amrani, 2020, El Bairi et al., 2020, El Bairi et al., 2017a, El Bairi et al., 2017b, El Bairi et al., 2021a, El Bairi et al., 2021b, https://www.springer.com/gp/book/9789811618727), one umbrella systematic review (El Bairi et al., 2021d), and several other ongoing original studies on OC without any funding or support. However, this is not enough to improve the management of OC patients and more interest for OC by the ministry of health and cancer organizations is awaited, particularly research that impacts clinical care. Collectively, we hope this correspondence will attract decision-makers and researchers to build research policies for this forgotten field.

## Declaration of Competing Interest

The authors declare that they have no known competing financial interests or personal relationships that could have appeared to influence the work reported in this paper.
